# Distinct Cholesterol Localization in Glioblastoma Multiforme Revealed by Mass Spectrometry Imaging

**DOI:** 10.1021/acschemneuro.2c00776

**Published:** 2023-04-11

**Authors:** Mai H. Philipsen, Ellinor Hansson, Auraya Manaprasertsak, Stefan Lange, Eva Jennische, Helena Carén, Kliment Gatzinsky, Asgeir Jakola, Emma U. Hammarlund, Per Malmberg

**Affiliations:** † Tissue Development and Evolution (TiDE) Division, Department of Laboratory Medicine, Lund University, SE22100 Lund, Sweden; ‡ Lund Stem Cell Center, Department of Laboratory Medicine, Lund University, SE22100 Lund, Sweden; § Sahlgrenska Centre for Cancer Research, Department of Medical Biochemistry and Cell biology, Institute of Biomedicine, Sahlgrenska Academy, University of Gothenburg, SE41390 Gothenburg, Sweden; ∥ Institute of Biomedicine, University of Gothenburg, SE41390 Gothenburg, Sweden; ⊥ Department of Neurosurgery, Sahlgrenska University Hospital, SE41345 Gothenburg, Sweden; # Institute of Neuroscience and physiology, Department of clinical neuroscience, Sahlgrenska Academy, SE41345 Gothenburg, Sweden; ∇ Department of Chemistry and Chemical Engineering, Chalmers University of Technology, SE41296 Gothenburg, Sweden

**Keywords:** mass spectrometry imaging, lipids, glioblastoma, cholesterol

## Abstract

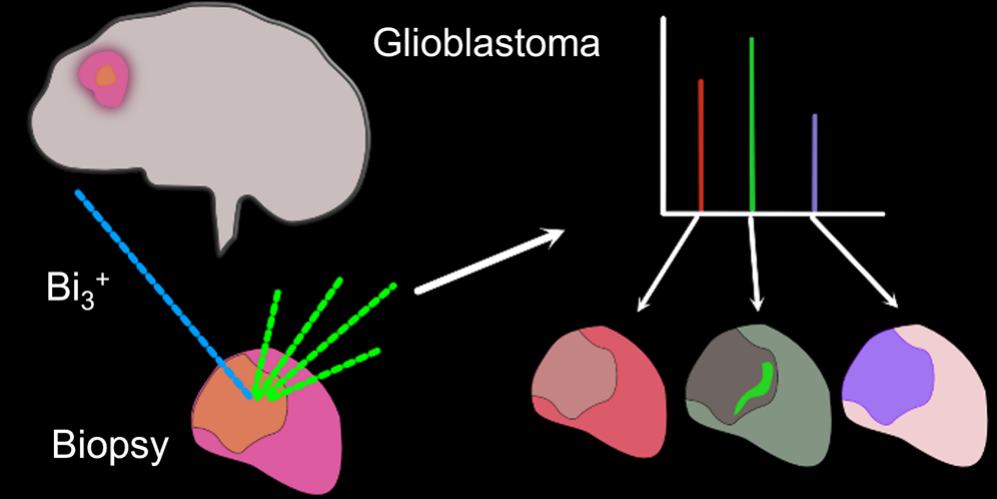

Glioblastoma multiforme (GBM) is the most common and aggressive brain tumor in adults and is highly resistant to chemo- and radiotherapies. GBM has been associated with alterations in lipid contents, but lipid metabolism reprogramming in tumor cells is not fully elucidated. One of the key hurdles is to localize the lipid species that are correlated with tumor growth and invasion. A better understanding of the localization of abnormal lipid metabolism and its vulnerabilities may open up to novel therapeutic approaches. Here, we use time-of-flight secondary ion mass spectrometry (ToF-SIMS) to spatially probe the lipid composition in a GBM biopsy from two regions with different histopathologies: one region with most cells of uniform size and shape, the homogeneous part, and the other with cells showing a great variation in size and shape, the heterogeneous part. Our results reveal elevated levels of cholesterol, diacylglycerols, and some phosphatidylethanolamine in the homogeneous part, while the heterogeneous part was dominated by a variety of fatty acids, phosphatidylcholine, and phosphatidylinositol species. We also observed a high expression of cholesterol in the homogeneous tumor region to be associated with large cells but not with macrophages. Our findings suggest that ToF-SIMS can distinguish in lipid distribution between parts within a human GBM tumor, which can be linked to different molecular mechanisms.

## Introduction

Cancer remains to be a major threat to human health. Glioblastoma multiforme (GBM) is one of the deadliest forms of cancer, with only a 5% chance of survival five years after diagnosis.^1^ While radiotherapy and chemotherapy marginally extend survival,^2^ the median survival after the diagnosis is between 10 and 19 months.^3−5^

Dysregulations of cellular metabolism are hallmarks of GBM, such as enabling replicative immortality, inducing angiogenesis, reprogramming cellular energetics, and evading immune destruction.^6^ Currently, it has been recognized that the evolution of GBM correlates with changes in lipid- and cholesterol-associated pathways.^7^ Malignant GBM associates with increased rates of lipid synthesis, hence leading to the elevation of intra-tumor concentrations of lipids, such as phosphatidylcholine (PC) and phosphatidylinositol (PI).^8^ Dysfunctional regulation of cholesterol is an indicator of most cancers, including that of glioblastoma.^9^ Previous work in connection between cholesterol dysfunction and glioblastoma concludes that there is a difference in the metabolism of cholesterol between healthy brain tissue and GBM tumors.^10,11^ Glioblastoma appears highly dependent on cholesterol for its survival.^12^

To advance our understanding of anomalies in lipid synthesis, mass spectrometry has been used to study of GBM tumors. Mass spectrometry unravels the chemical composition of any biological tissues.^13−15^ In particular, mass spectrometry imaging (MSI) is useful for visualizing the variation in chemical composition across a tumor sample. MSI works by identifying the mass over charge ratio (*m*/*z*) of each molecular species that enters the mass analyzer. In addition, MSI spatially connects each peak in the mass spectrum to the areas of the sample from which it originates and, thus, identifies the originating location of that species. Chemical mapping is useful to capture the distribution of different molecules throughout the tissue, especially for understanding heterogeneous tissue, such as a tumor.

Among MSI techniques, time-of-flight secondary ionizing mass spectrometry (ToF-SIMS) has the potential to track down the spatial distribution of metabolites, lipids, or elements in tissues to the subcellular scale. ToF-SIMS is therefore suitable to explore the role of cholesterol during GBM tumor maintenance and progressions. Recently, ToF-SIMS successfully visualized the different distributions of biomolecules, e.g., glutamine and cholesterol, between transformed (glioblastoma) and nontransformed tissues.^13^ This work highlights how well MSI can capture the specific distribution of cholesterol within the transformed tissue. The specific distribution of cholesterol within the transformed tissue can be expanded upon, not the least since the samples used in the study were embedded in optimal cutting temperature medium (OCT) prior to freezing. OCT contains poly(vinyl alcohol) and poly(ethylene glycol), which can alter the chemical distributions, cause ion suppression, and contaminate the sample. It is therefore of particular importance that further studies using MSI also clarify the chemical landscape of pristine GBM samples.

Here, we perform chemical imaging analyses of human glioblastoma using ToF-SIMS, with the aim to map out the lipid profiles in two different GBM tumor regions. To reduce the risk of negative effects of embedding, we used the fresh frozen samples without embedding medium.

## Results and Discussion

ToF-SIMS was used to analyze the lipid distribution from two different GBM regions including the homogeneous and heterogeneous regions. These regions have been identified by hematoxylin and eosin (H&E) staining of the sections (Figure S1). The biopsy had two distinctive parts, from two regions with different histopathologies: one with most cells of uniform size and shape, the homogeneous part, and the other with cells showing a great variation in size and shape, the heterogeneous part, with a distinct border between them. The heterogeneous part (marked with a circle in Figure S1) was composed of cell types with a great variation in size and shape. Most of the cells showed expression of glial fibrillary acidic protein (GFAP), but groups of cells were GFAP negative. In the homogeneous part, most of the cells were of a similar size and shape and expressed GFAP patterns.

Prior to ToF-SIMS experiments, frozen GBM sections were freeze-dried and then analyzed with Bi_3_^+^ primary ion gun under the vacuum condition. Thereafter, the distribution of different lipids from ToF-SIMS images was correlated with the same histological regions from the H&E staining image to obtain useful information of GBM behavior and development. It is noted that the use of gas cluster ion beams (Bi_3_^+^) with high energy causes the fragmentation of biomolecules. Hence, the high intensities of lipid fragments can be achieved. In contrast, intact lipid molecules such as PC, diacylglycerol (DAG), phosphatidylethanolamine (PE), and PI can be detected, but their intensities were low. Both fragments and intact molecules of several lipids are distributed differently in the homogeneous part compared with those in the heterogeneous part.

**Figure 1:**
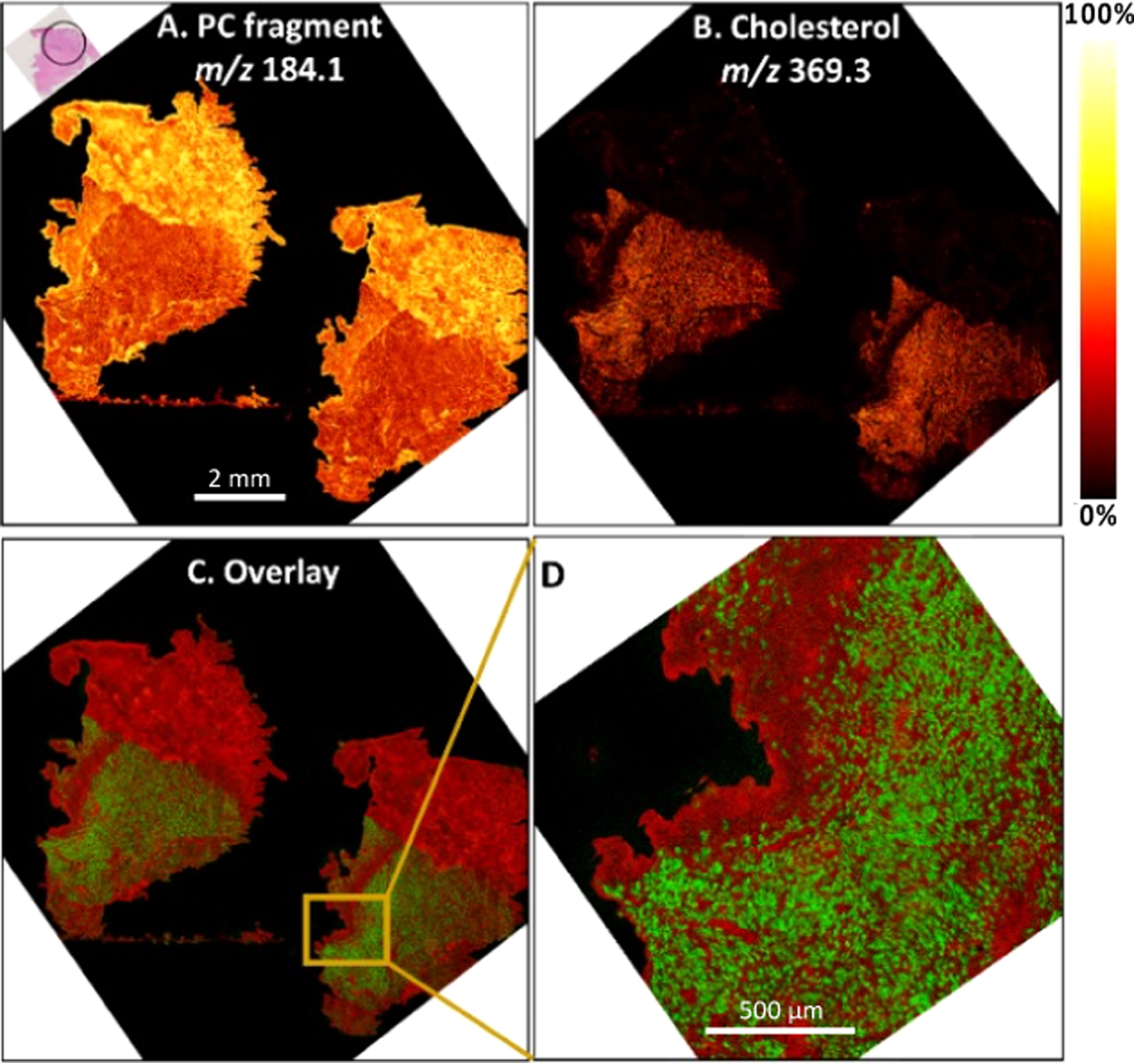
ToF-SIMS ion images of a PC fragment at *m*/*z* 184.1 (A) and cholesterol at *m*/*z* 369.3 (B) were observed by ToF-SIMS in the positive ion mode. Overlay with cholesterol in green and PC in red (C) and a closeup in (D). Scale bar is 2 mm. The thermal scale is shown to the right of the image. The H&E staining image in the top left illustrates different regions of tumor, including the heterogeneous part (marked in a black circle) and the homogeneous part.

ToF-SIMS images show the opposite changes in the distribution of cholesterol at *m*/*z* 369.3 and one PC fragment at *m*/*z* 184.1 between the homogeneous and heterogeneous regions, as shown in Figure 1. Both molecules are found over the entire tumor sections. However, cholesterol is more abundant in the homogeneous part compared with the heterogeneous part, whereas PC fragments are more abundant in the heterogeneous part of tumor compared with the homogeneous part. However, sphingomyelin (SM) also represents phosphocholine headgroup in its structure similar to PC; the *m*/*z* 184 peak might result from either PC or SM. In this study, the fragment of PC at *m*/*z* 224 distinct from SM changed similarly to *m*/*z* 184 between different samples (Figure S10), thus suggesting that the change for *m*/*z* 184 is more likely or at least in part from the phosphocholine headgroup. A magnified image of the distribution of cholesterol and PC fragments is displayed in Figure 1D, in which cholesterol can be seen aggregating in a pattern similar in size and shape to cells in the homogeneous regions. In accordance to this, ion images of cholesterol at *m*/*z* 368.3 in the negative ion mode show the enhancement in the homogeneous tumor area compared with the heterogeneous part (Figure S2). In post contrast, the [CN]® peak at *m*/*z* 26.0, a fragment from proteins and nucleic acids,^16^ is more abundant in the heterogeneous tumor region. The difference in distribution of cholesterol correlates to the pathologically confirmed different parts of the tumor. In the homogeneous region, the cholesterol appears to reside entirely within the cells, indicating the loading of cholesterol as part of cancer cell metabolism.^12,17^

In normal human tissues, cholesterol is a major component of myelin sheaths and produced via *de novo* biosynthesis mainly in glial cells.^18^ Cholesterol plays a key role in cellular processes by maintaining the rigidity of cell membranes and providing a medium for signaling transduction.^19^ Over the past few decades, several studies have found high cholesterol contents in transformed tissue, such as that of glioblastoma.^8,20−22^ However, the increase is not necessarily a result of increased synthesis. In GBM, cells elevate uptake of exogenous lipids and lipoproteins rather than stimulate cholesterol synthesis.^11,23^ Also, the downregulation of enzymes that otherwise degrade cholesterol is associated with GBM. A study conducted by Han et al. reported a reduction of cholesterol 24-hydroxylase in GBM cells *in vivo*,^24^ which was argued responsible for the accumulation of excessive intracellular cholesterol to support the tumor growth.

**Figure 2:**
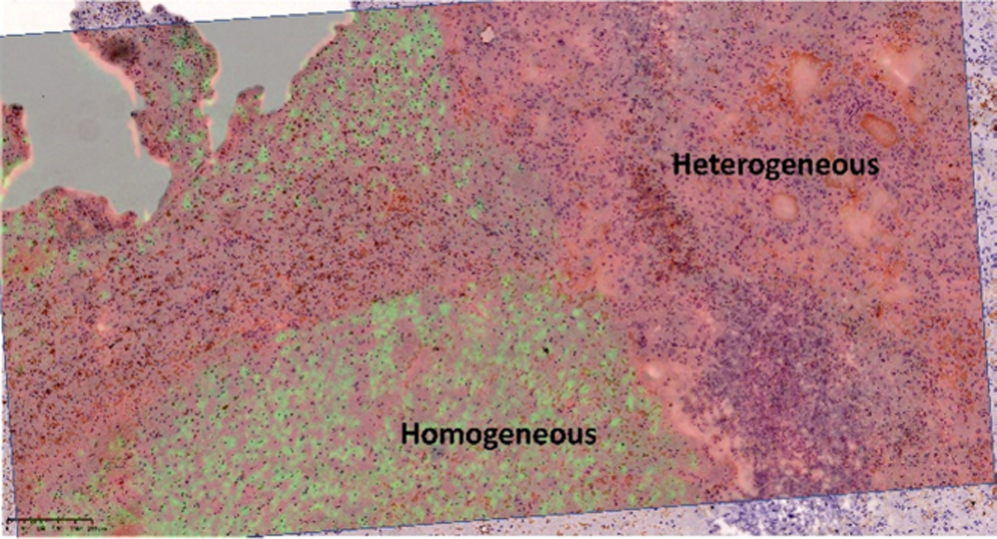
Distribution of cholesterol and macrophages in GBM tissue. A ToF-SIMS pseudocolored image represents cholesterol at *m*/*z* 369.3 (green) and a PC fragment at *m*/*z* 184 (red). The sections analyzed by ToF-SIMS were stained with CD68 against macrophages (brown). The scale bar is 250 μm.

We found the high levels of cholesterol in the homogeneous part of the biopsy. This excess of intracellular cholesterol is usually stored in lipid droplets as cholesterol esters, and generally accumulation of lipid droplets is observed in a variety of cancer cells, especially in GBM.^25−28^ Recent studies have shown that a large number of lipid droplets are detected in tumor tissues from GBM patients, but not in the normal brain tissues and low-grade gliomas.^29^ Moreover, lipid droplets in cancer cells are also involved in the polarization of tumor-associated macrophages (TAMs).^30^ TAMs dominate the population of immune cells in the GBM tumor microenvironment.^31−33^ These cells enhance tumor cell invasion, motility, and intravasation in primary tumor. During metastasis, TAMs stimulate the tumor growth, extravasation, and survival.^34^ We hypothesize that TAMs might contain a large amount of lipid droplet-stored cholesterol in human GBM tumor. To test our hypothesis, the same tissue sections as imaged by ToF-SIMS were stained subsequently with CD68 of macrophages (Figure 2). Cells positive for the macrophage marker CD68 were more abundant and more varied in size and shape in the heterogeneous part of the biopsy than in the homogeneous part. Notably, the bombardment of primary ion beams during TOF-SIMS analysis did not appear to have an effect on cell morphology. Post-ToF-SIMS staining of the tissue showed that cell morphology was intact and that the distribution of CD68-positive cells was similar as in the sections not exposed to TOF-SIMS (Figure S2). Thus, we concluded that cholesterol was enriched in the homogeneous tumor regions, while macrophages are more abundant in the heterogeneous parts. The overlay image (Figure 2) of two methods illustrates that cholesterol localization is correlated with some cells in the homogeneous tumor regions, but not with the macrophages.

**Figure 3:**
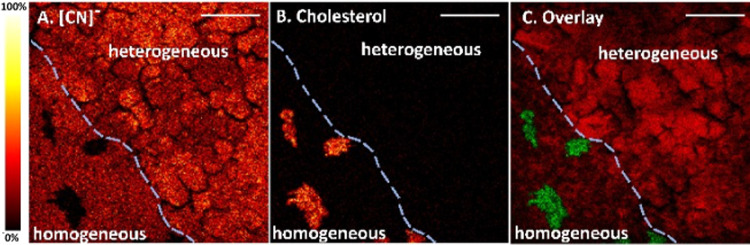
ToF-SIMS ion images in the negative ion mode of the interface between homogeneous tumor and heterogeneous tumor regions divided by the dashed line; scale bar is 50 μm. (A) [CN]® at *m*/*z* 26.0, (B) cholesterol at *m*/*z* 368.3, and (C) an overlay of [CN]® (red) and cholesterol (green). The spatial resolution is approximately 500 nm. The thermal scale bar is shown on the left of the image.

To closely examine the cellular structure of the cholesterol in the homogeneous tumor region, a more detailed and high-resolution analysis was conducted using the delayed extraction mode on the ToF-SIMS (Figure 3). This shows that the cholesterol in the homogeneous parts of the biopsy localizes in cell-sized structures, as shown in Figure 3B. Similar to our findings, Gularyan et al. found the co-localization of a small population of cells and cholesterol in human GBM tissue using ToF-SIMS.^35^ Using the glioblastoma stem cell marker, CD133, the authors proposed that these cells enriched in cholesterol may represent glioblastoma stem cells. This finding would support the hypothesis that cancer stem cells can stimulate cholesterol biosynthesis, which in turn causes the increase in cholesterol levels.

**Figure 4:**
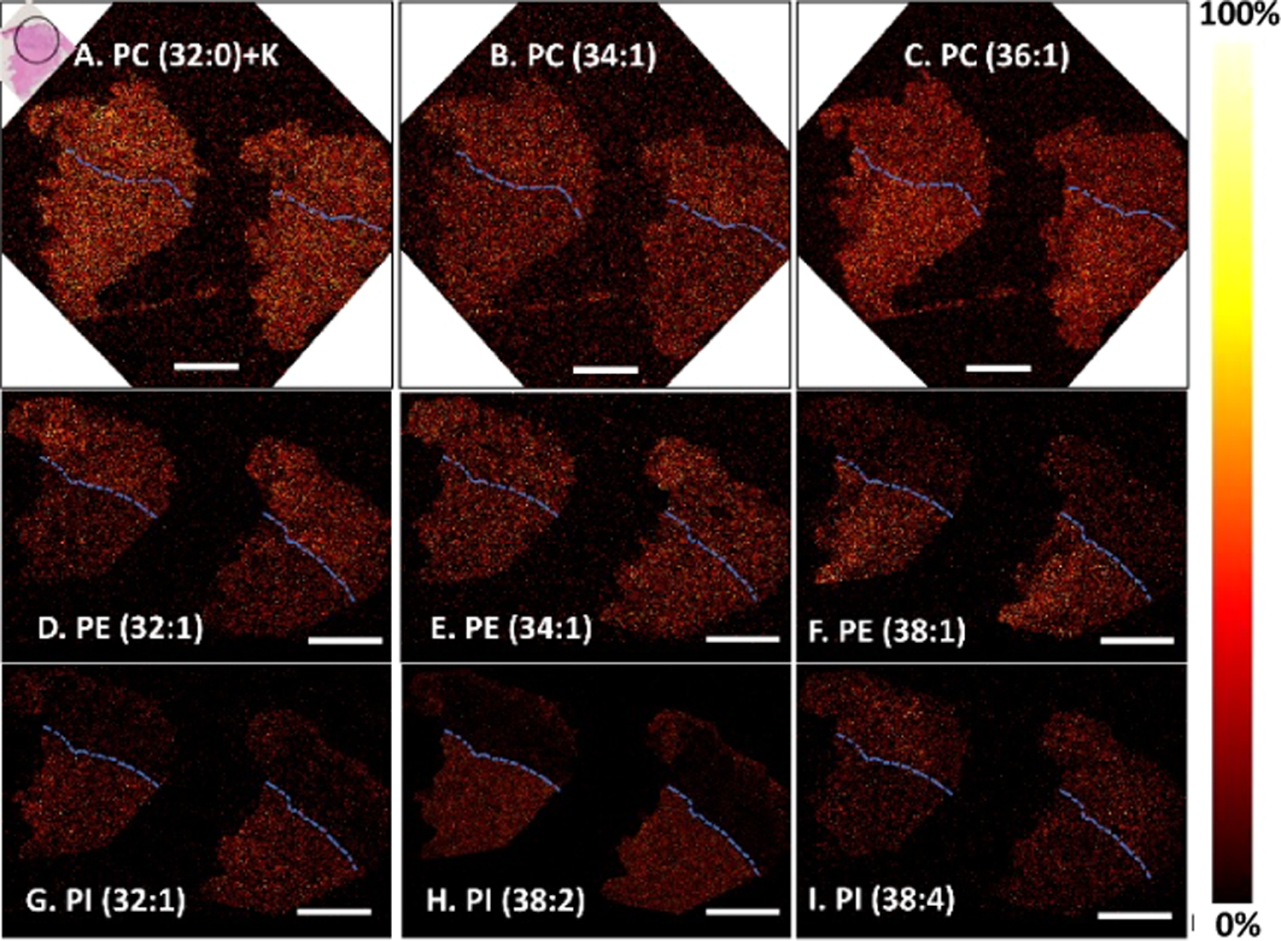
ToF-SIMS ion images show the localization of different lipid species in the heterogeneous tumor (marked in a black circle in the H&E staining image) and homogeneous regions. (A–C) PCs in the positive ion mode, (D–F) PEs, and (G–I) PIs in the negative ion mode. Note that unsaturated lipids with longer carbon acid chains including PC (36:1), PE (38:1), and PI (38:2) display higher intensity in the homogeneous area of GMB biopsy. Scale bar is 2 mm. The blue dashed line displays the border between homogeneous tumor and heterogeneous region. The thermal scale is shown on the right of the image.

The enhancement of cholesterol in glioblastoma cells could be adjoined by amplified synthesis of several other lipids in the cell. We, therefore, examine another lipid, known as phospholipids, including PC, PE, and PI in the GBM tissues. Recent work demonstrated that phospholipid levels, such as PCs, in GBM tumor tissue were to be higher than those in nontransformed tissues.^8,21^ Similar to this finding, most previous studies have investigated the alteration of phospholipids in transformed (GBM) versus nontransformed tissues. For the first time, we here show the alteration of lipid localization within the (human) GMB and between its different regions. Our ToF-SIMS data demonstrate the diverse trends in the distributions of intact lipids including PCs, PEs, and PIs in the tumor tissue (Figure 4). Phospholipids with different lengths of carbon acid chains are located in different parts of the tumor. For example, some PC, PE, and PI species, such as PC (34:1) at *m*/*z* 760.6, PE (32:1) at *m*/*z* 688.5, and PI (38:4) at *m*/*z* 885.5, are uniform across the entire tissue but appear a little stronger in the heterogeneous tumor regions. In contrast, other phospholipids including PC (36:1) at *m*/*z* 788.6, PE (38:1) at *m*/*z* 772.6, PI (32:1) at *m*/*z* 807.5, and PI (38:2) at *m*/*z* 889.6 are highly distributed the homogeneous tumor part. An exception is that PC (32:0) + K at *m*/*z* 772.5 and PE (34:1) at *m*/*z* 716.5 are distributed evenly across two tumor regions. We found that most of unsaturated lipids with longer carbon acid chains including PC (36:1), PE (38:1), and PI (38:2) display higher intensity in the homogeneous area of GMB biopsy. Since the phospholipid composition appears a deciding factor in the proliferation of glioma, a high amount of PE in the cellular membrane is argued necessary for continuous cell division.^36^ However, the molecular mechanism leading to an alteration in phospholipid composition remains unknown.

Recently, the overexpression of proteins regulating lipid metabolisms, which might induce an increase in lipid levels, has been demonstrated in malignant gliomas.^36,37^ For instance, phosphatidylethanolamine-binding protein 4 (PEBP4), initially bound to PEs, plays a vital role in cancer progression including invasion and metastasis.^38,39^ In accordance with this, PEBP4 has been found to be highly expressed in several cancers,^38,40,41^ including GBM,^36^ which might result in the increase in PE levels in GBM tissues. In addition, phospholipid synthesis can be regulated by fatty acids, important precursors for lipid synthesis, in glioblastoma tissues.^42,43^

**Figure 5:**
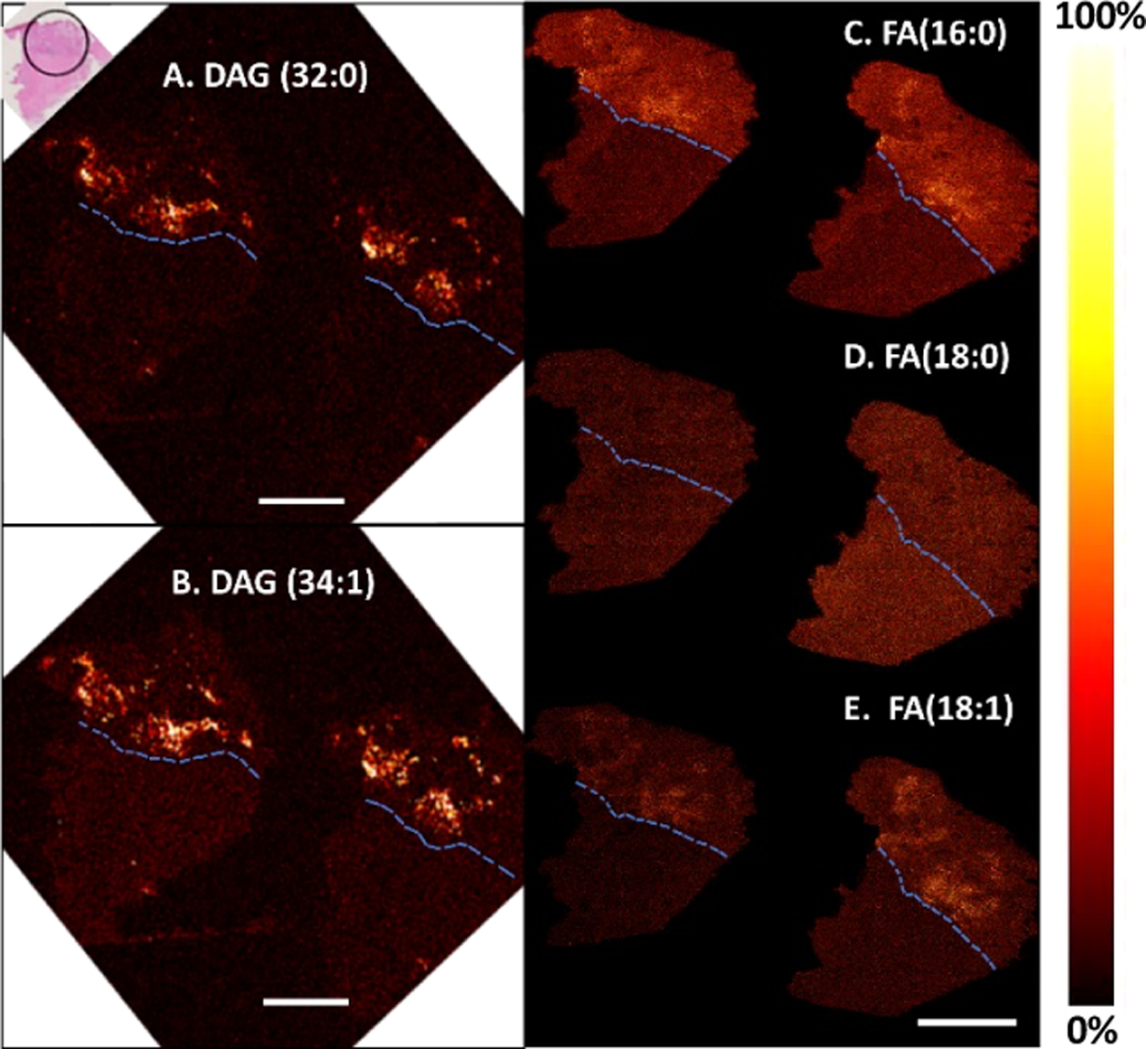
Distribution of DAGs in the positive ion mode and fatty acids in the negative ion mode in the tumor. (A) DAG (32:0) at *m*/*z* 551.5, (B) DAG (34:1) at *m*/*z* 577.5, (C) FA (16:0) at *m*/*z* 255.2, (D) FA (18:0) at *m*/*z* 283.2, and (E) FA (18:1) at *m*/*z* 281.2. All data were acquired using ToF-SIMS equipped with a 25 keV Bi_3_^+^ ion beam. Scale bar is 2 mm. The dashed line displays the border between the homogeneous and heterogeneous regions. The H&E staining image (top left) illustrates the heterogeneous tumor region, marked in a black circle, and the homogeneous part of the tumor. The thermal scale is shown on the right of the image.

In the next step, we examined the distributions of fatty acids in GBM tissues, as shown in Figure 5. ToF-SIMS data display that fatty acids are located in the whole tissue sample. Also, we found that the accumulation of fatty acids occurred at the interface between the homogeneous part of the biopsy and the heterogeneous part, which was associated with the localization of diacylglycerols (DAGs). In Figure 5, DAGs and some fatty acids are predominantly located in the heterogeneous tumor and around the interface. The distribution of DAG species in positive ion mode, such as DAG (32:0) at *m*/*z* 551.5 and DAG (34:1) at *m*/*z* 577.5, at the interface is correlated with the distribution of fatty acids (FAs) in negative ion mode including FA (16:0) at *m*/*z* 255.2 and FA (18:1) at *m*/*z* 281.2. Saturated FAs (18:0), however, are more evenly distributed in different tumor regions. This distribution is not previously documented in the literature, and while DAG has been analyzed with MS previously, this, to the best of our knowledge, is the first identification of DAG at the interface of two tumor regions. It should be noted that FAs and DAG detected by ToF-SIMS could also be fragments of triacylglycerol (TAG).^44^ A recent study demonstrated that FA (18:1) induced the accumulation of lipid droplet-enriched TAGs in GBM cell lines that promote GBM proliferation.^27^ The authors suggested that fatty acids increase proliferation of GBM cells via TAG metabolism. In our study, the co-localization of both fatty acids and DAGs found at the border of two tumor regions might suggest that GBM tumor can regulate their lipid composition by increasing TAG accumulation to support their growth and invasion.

## Conclusions

The difficulty to effectively treat GBM tumor largely depends on its invasiveness and treatment resistance.^38^ Accordingly, studying the progression of the tumor and the underlying pathophysiological mechanism is of great interest. A difference in lipid metabolism is a common denominator for many cancers.^45−47^ While the focus of the study of tumor metabolism usually lies in carbohydrates, accumulating observations point to the importance of lipid metabolism for the survival and growth of a tumor, especially in human GBM.^35,48^ Altered lipid metabolism has been identified as a metabolic feature of GBM stem-like cells, linking lipid metabolism to treatment resistance and relapse.^35^ Hence, an advanced understanding of the role of lipids in GBM may shed light on the novel targets.

Using ToF-SIMS, we further confirmed the differences in the distribution of several lipids including cholesterol, PCs, PEs, PIs, DAGs, and fatty acids between two different tumor regions of GBM tissues. We found that cholesterol was accumulated mainly in the homogeneous region compared with the heterogeneous part of the tumor. This cholesterol loading may indicate the need of cholesterol in tumor development and invasion of the surrounding normal brain tissue. Furthermore, the correlation of ToF-SIMS and staining for macrophages also confirmed that the increase in cholesterol occurs in the cells which was not associated with macrophages and could therefore be associated with, e.g., glioblastoma stem cells or necrotic areas. Similar to cholesterol loading, some phospholipids including PC, PE, and PI with long carbon acid chains had high abundance in the homogeneous region. However, fatty acids, phospholipid precursors, were highly localized in the heterogeneous region and accumulated at the border between the two areas. Especially, fatty acids were associated with DAGs, fragments of TAGs, which indicated that the mechanism involved the loadings of TAGs in the heterogeneous part. In summary, the distribution of lipids varies between two different regions of the GBM tumor tissues. These changes might be involved in different molecular mechanisms related to tumor development and invasion. Hence, the chemical mapping of lipids might open opportunities for novel GMB treatment options.

## Methods

### Chemicals

All chemicals were purchased from Sigma–Aldrich, Sweden.

### GBM Tissue Sampling and Sectioning

The human tumor biopsy was handled under the ethical permit with number 604-12. A grade IV glioblastoma tumor (diameter of 6 cm), located temporally on the right side, was removed with primary surgery. The tumor was MGMT unmethylated, demonstrating high contrast and central necrosis. A small part of the tumor was sampled during regular tumor resection following informed consent. The tissue sample from one patient was freshly frozen in a −80°C freezer and kept frozen until use. Cryostat sections were cut at 8 μm and placed on superfrost plus microscope slides. Sides for TOF-SIMS experiments were kept at −80°C until use.

### Post-ToF-SIMS H&E and CD68 Staining

Serial sections were fixed with methanol and stained with H&E stain for general morphology. Further sections were used for immunohistochemical expression of CD68 (DAKO). In short, the sections were fixed in methanol, blocked with goat serum and incubated at 5 °C overnight with the primary antibody. The ImmPRESS-HRP anti-rabbit IgG polymer detection kit, made in goat, or the ImmPRESS-HRP anti-mouse IgG polymer detection kit, made in horse (Vector Laboratories), were used as secondary reagents for rabbit or mouse primary antibodies, respectively. The immunoreaction was visualized using the DAB+ substrate chromogen system (DAKO). Nuclei were counterstained with hematoxylin, and the slides were dehydrated and mounted in DPX (Merck).

In order to the check if the morphology of the sections had been affected by the TOF-SIMS procedure, the slides exposed for TOF-SIMS experiments were stained to visualize CD68-positive cells as described above.

### ToF-SIMS Analysis

ToF-SIMS analyses were performed on six tumor sections under static SIMS conditions using a TOF.SIMS V (ION-TOF GmbH, Münster, Germany) equipped with a 25 KeV bismuth (Bi_3_^+^) liquid ion gun as a primary ion. Images were recorded in both positive and negative ion modes. The current of the primary ion was 0.26 pA with a maximum ion dose of 1 × 10^11^ ions/cm^2^. Large area analysis was performed using the raster stage scan mode with two shots per pixel to cover the entire tissue area, using a pixel resolution of about 3 μm/pixel. The delayed extraction mode was used for single cell imaging with the high spatial resolution of about 500 nm while maintaining high mass resolution of 3000 at *m*/*z* 58. Images of 150 × 150 μm^2^ with 256 × 256 pixels were acquired. All ToF-SIMS spectra and images were acquired, processed, and analyzed using the SurfaceLab 7 software (version 7.0 ION-TOF, GmbH). The mass spectra were internally calibrated to signals of [C]^+^, [CH_2_]^+^, and [CH_3_]^+^, for the positive ion mode, while common fragment peaks at [C]^−^, [CH]^−^, [C_2_]^−^, and [C_3_]^−^ were used for calibration in the negative ion mode.

## Supporting Information Available

Microscopic images of H&E staining sections of GBM tissues, microscopic images of CD68-staining GBM sections after analyzing with ToF-SIMS, and ToF-SIMS ion images in the negative ion mode (PDF)

## Abbreviations

GBM: glioblastoma multiforme
MSI: mass spectrometry imaging
ToF-SIMS: time-of-flight secondary ion mass spectrometry
PE: phosphatidylethanolamine
PC: phosphatidylcholine
PI: phosphatidylinositol
DAG: diacylglycerol
TAG: triacylglycerol
FA: fatty acid
TAM: tumor-associated macrophages
H&E: hematoxylin and eosin
GBM: glioblastoma multiforme
PEBP4: phosphatidylethanolamine-binding protein 4
GFAP: glial fibrillary acidic protein
