# Supplementary material for: Distinct Cholesterol Localization in Glioblastoma Multiforme Revealed by Mass Spectrometry Imaging
Source: ACS Chem Neurosci. 2023 Apr 11;14(9):1602–9. doi: 10.1021/acschemneuro.2c00776 (PMC10161228; doi:10.1021/acschemneuro.2c00776)
Supplement: Supplementary file 1 — cn2c00776_si_001.pdf [file cn2c00776_si_001.pdf]

Supporting Information

Distinct cholesterol localization in glioblastoma multiforme revealed by mass spectrometry imaging

Mai H. Philipsen[†‡], Ellinor Hansson[*], Auraya Manaprasertsak[†‡], Stefan Lange[⊥], Eva Jennische[⊥], Helena Carén[⊥], Kliment Gatzinsky[‖], Asgeir Jakola[‖], Emma U. Hammarlund[†‡] and Per Malmberg[*]

[†] Tissue Development and Evolution (TiDE) Division, Department of Laboratory Medicine, Lund University, SE22100 , Lund, Sweden

[‡] Lund Stem Cell Center, Department of Laboratory Medicine, Lund University, SE22100, Lund, Sweden

[y] Sahlgrenska Centre for Cancer Research, Department of Medical Biochemistry and Cell biology, Institute of Biomedicine, Sahlgrenska Academy, University of Gothenburg, SE41390, Gothenburg, Sweden.

[⊥] Institute of Biomedicine, University of Gothenburg, SE41390, Gothenburg, Sweden

[‖] Department of Neurosurgery, Sahlgrenska University Hospital, SE41345, Gothenburg, Sweden

[ꜰ] Institute of Neuroscience and physiology, Department of clinical neuroscience, Sahlgrenska Academy, SE41345, Gothenburg, Sweden

[*] Department of Chemistry and Chemical Engineering, Chalmers University of Technology, SE41296,Gothenburg, Sweden

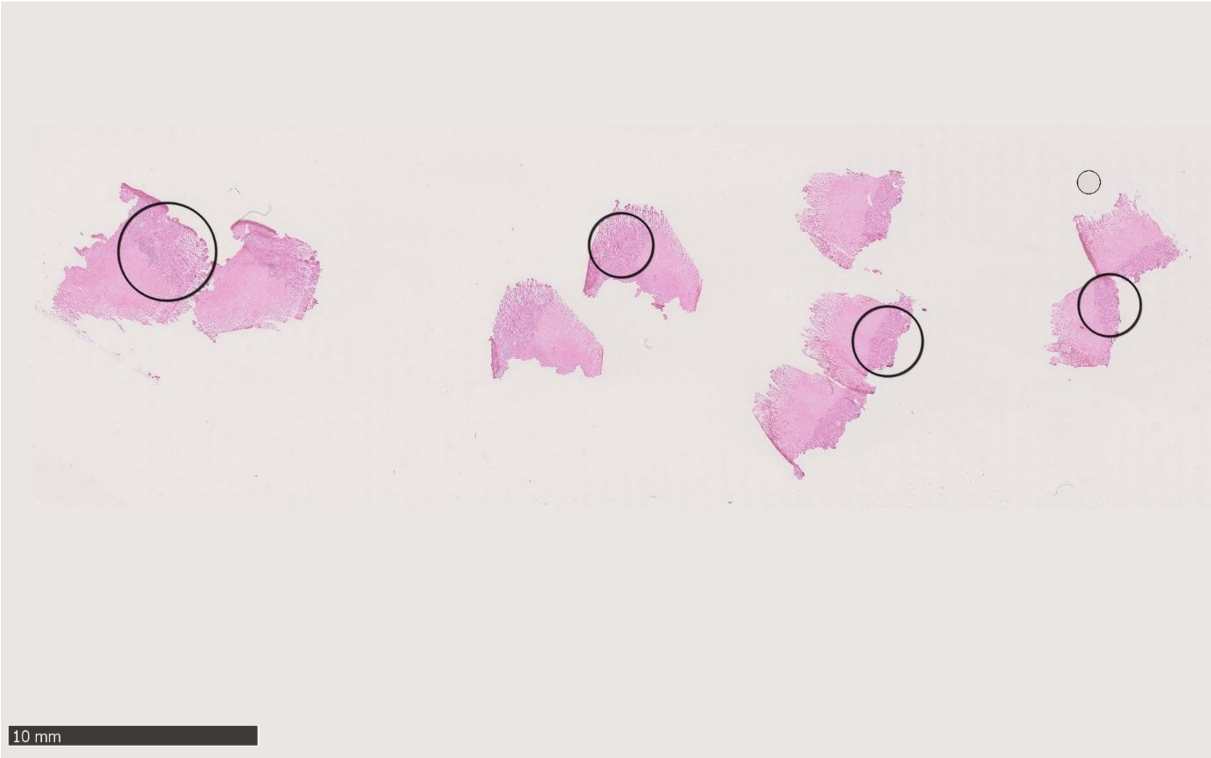

Figure S1. Microscope images of hematoxylin eosin staining sections of GBM tissues. The dark purple areas marked in black circles are corresponded for the central tumor parts while the lighter purple regions are the infiltrating tumor parts. The scale bar is 10 mm.

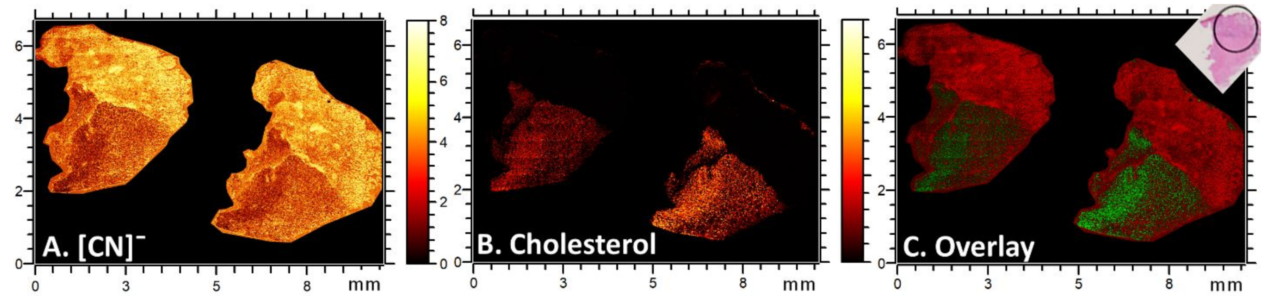

Figure S2. ToF-SIMS ion images of $[CN]^-$ at *m/z* 26.0 (A) and cholesterol at *m/z* 368.3 (B) were observed by ToF-SIMS in the negative ion mode. Overlay with cholesterol in green and $[CN]^-$ in red (C). The thermo scale is shown to the right of the image. The H&E staining image in the top right illustrates the inner tumorregion, marked in black ring, and the infiltrating part of the tumor.

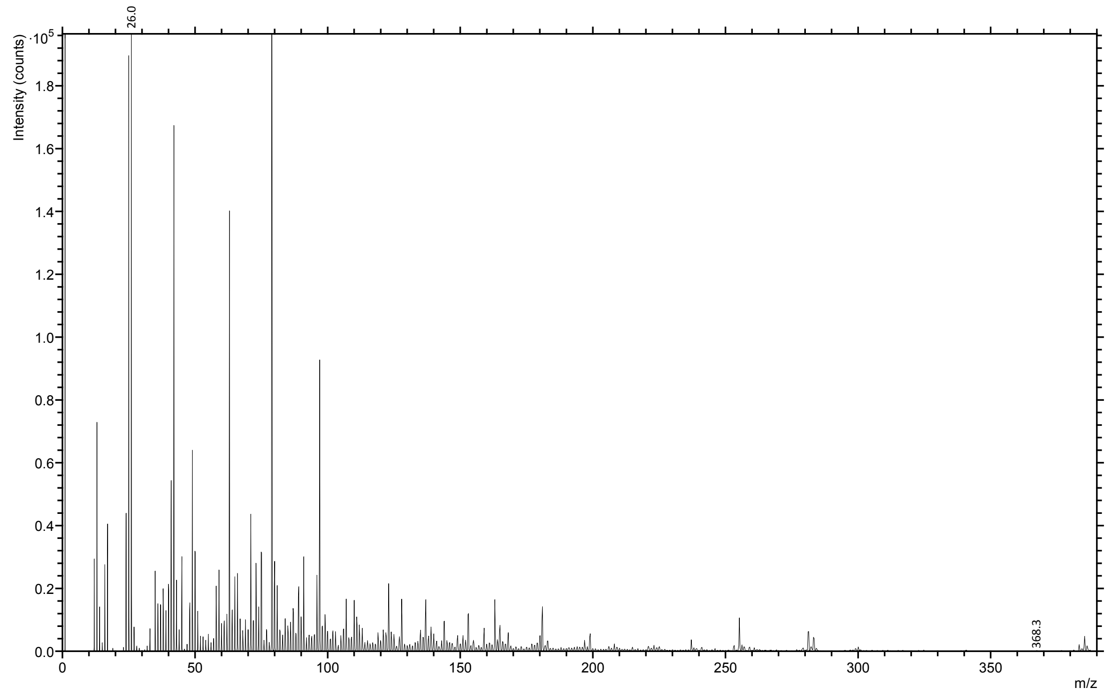

Figure S3. ToF-SIMS spectrum in the negative ion mode in a mass range of m/z 700-900 of the interfaces between homogeneous tumor and heterogeneous tumor regions. The peaks at m/z 26.0 and 368.3 represent cyanide $[CN]^-$ and cholesterol fragment $[C_{27}H_{44}]^-$, respectively. The spectrum corresponded to ToF-SIMS images in Figure 3.

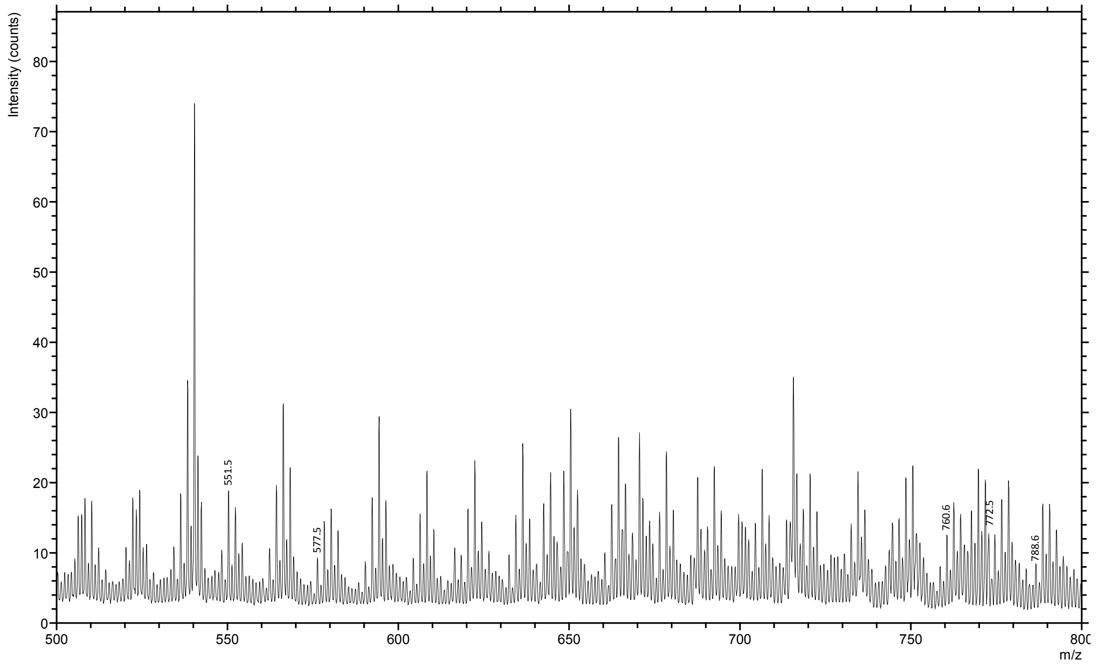

Figure S4. ToF-SIMS spectrum in the positive ion mode in a mass range of m/z 500-800 of glioblastoma tissue sections. The peaks at m/z 772.5, 760.6 and 788.6 represent PC (32:0)+K, PC (34:1) and PC (36:1), respectively. The peaks at m/z 551.5 and 577.5 represent DAG (32:0) and DAG (34:1), respectively.

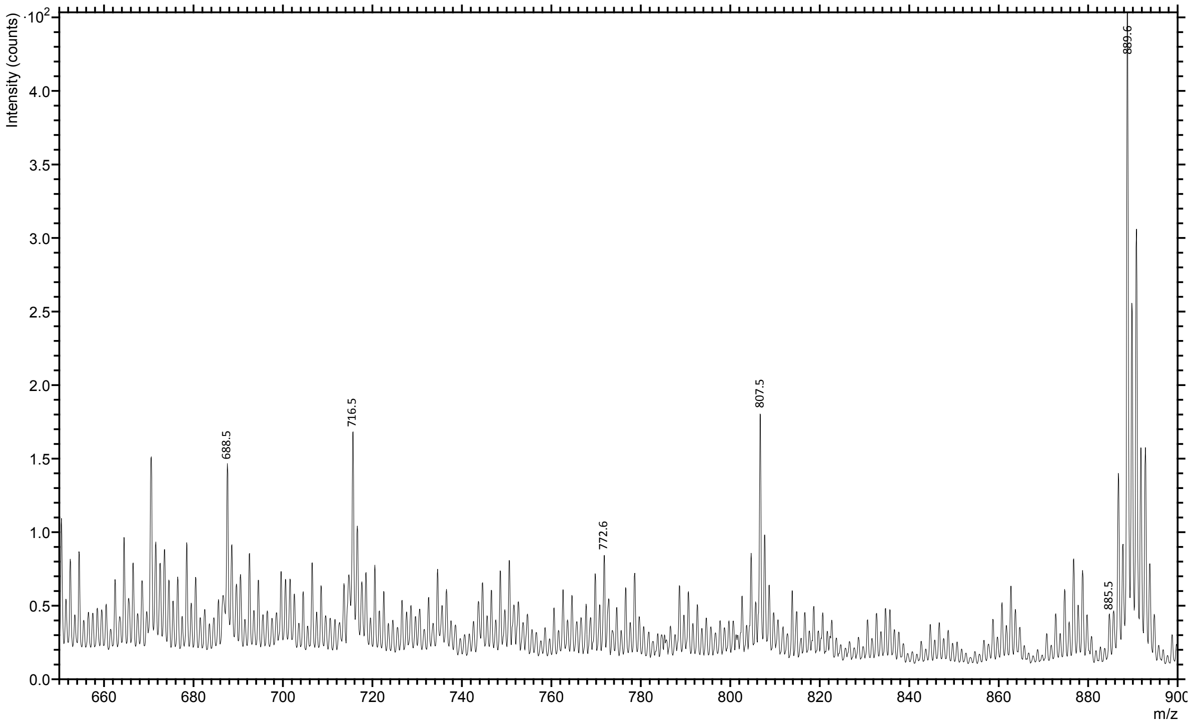

Figure S5. ToF-SIMS spectrum in the negative ion mode in a mass range of m/z 650-900 of glioblastoma tissue sections. The peaks at m/z 688.5, 716.5 and 772.6 represent PE (32:1), PE (34:1) and PE (38:1), respectively. The peaks at m/z 807.5, 889.6 and 885.5 represent PI (32:1), PI (38:2) and PI (38:4), respectively.

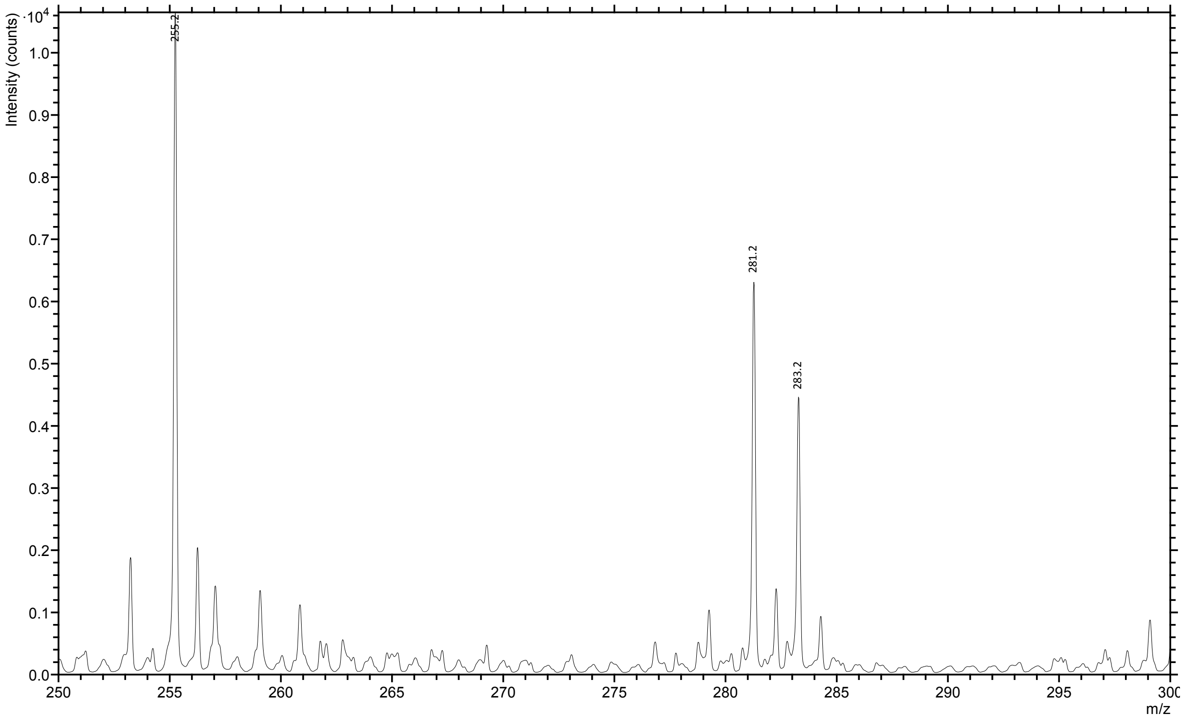

Figure S6. ToF-SIMS spectrum in the negative ion mode in a mass range of m/z 250-300 of glioblastoma tissue sections. The peaks at m/z 255.2, 283.2 and 281.2 represent FA (16:0), FA (18:0) and FA (18:1), respectively.

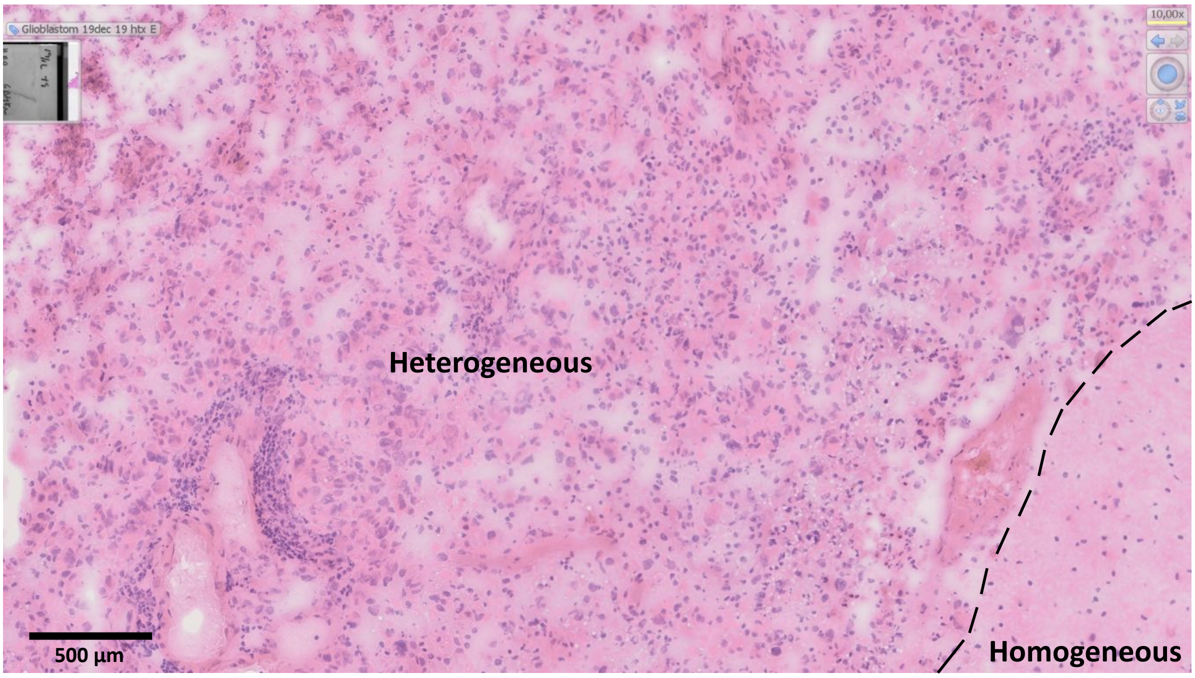

Figure S7. Microscope image of hematoxylin eosin staining section of GBM tissue. The interface between homogeneous and heterogeneous tumor regions divided by dash line.

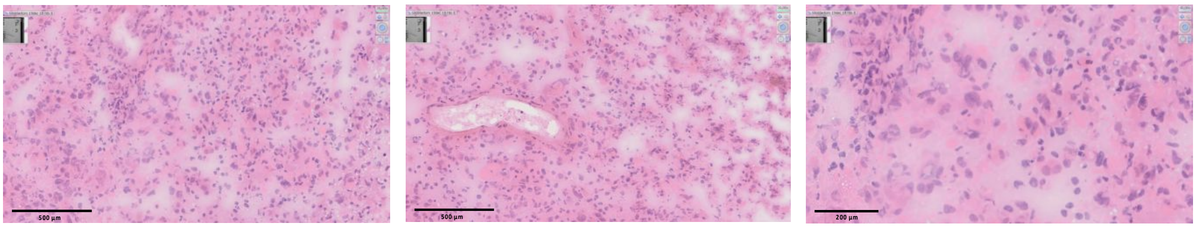

Figure S8. Microscope images of hematoxylin eosin staining sections of GBM tissue in the heterogeneous tumor region.

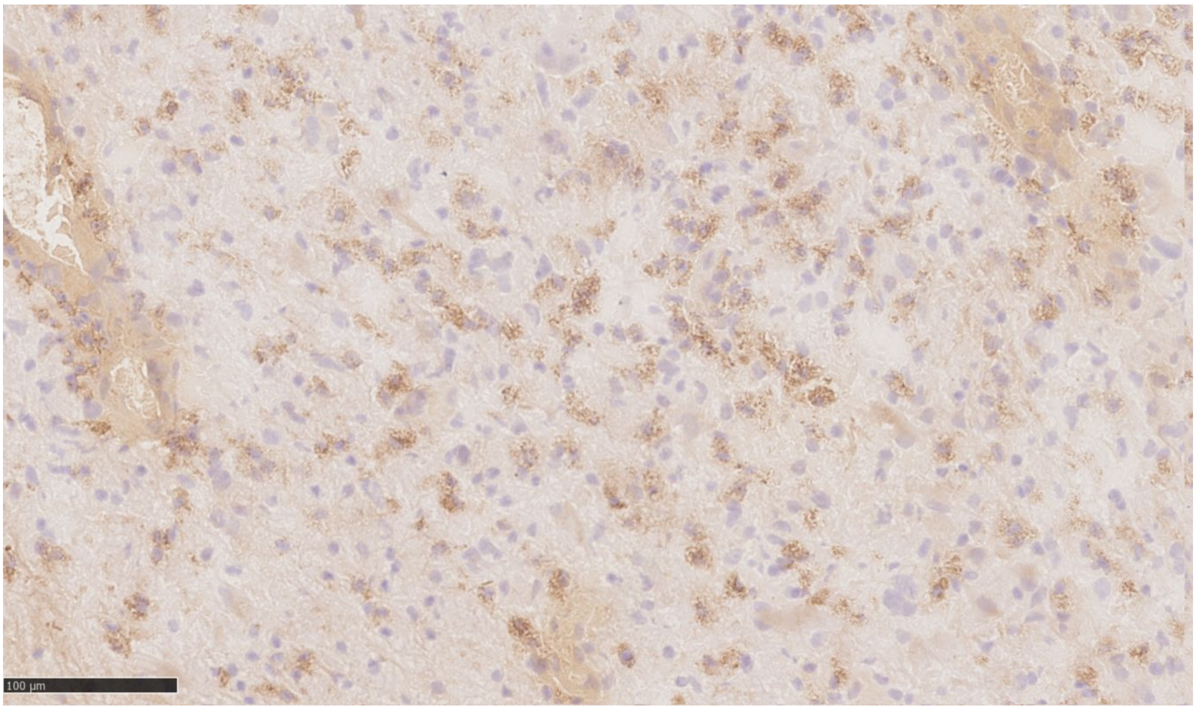

Figure S9. Microscope image of the section analyzed by ToF-SIMS were stained with CD68 against macrophages.

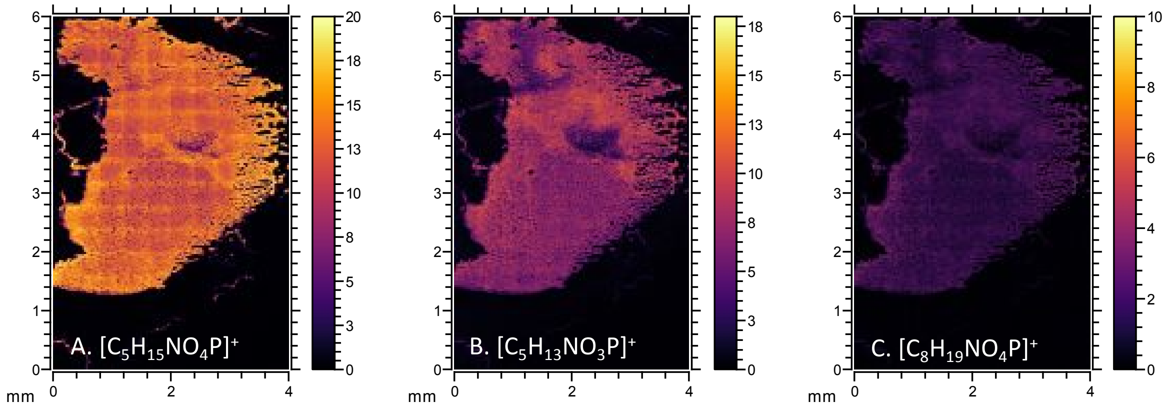

Figure S10. ToF-SIMS ion images of $[C_5H_{15}NO_4P]^+$ at *m/z* 184.1 (A), $[C_5H_{13}NO_3P]^+$ at *m/z* 166.1 (B) and $[C_8H_{19}NO_4P]^+$ at *m/z* 224.1 (C) were observed by ToF-SIMS in the positive ion mode.
